# Supplementary material for: Physical exercise and chronic pain in university students
Source: PLoS One. 2020 Jun 26;15(6):e0235419. doi: 10.1371/journal.pone.0235419 (PMC7319292; doi:10.1371/journal.pone.0235419)
Supplement: Attachment — Submitted filename: renamed_f125d.docx [file pone.0235419.s001.docx]

**Re: comments on the manuscript number “**PONE-D-20-09746**”.**

**Importance and objective of the paper**

Chronic pain is a major health problem globally. It is highlighted as one of the leading cause of years lived with disability, imposing substantial burdens on individuals and community. Although there are small percent of people develop chronic pain, the treatment for those people are complicated and costly. The beneficial effects of physical activity for many chronic diseases have been well documented. Therefore, I see this article is important as it investigates the association between engaging in different amounts of physical exercise and chronic pain.

Generally, the authors do a good job in describing the problem and research question, detailing the methods and results, and providing contextual information to aid in the interpretation of the results. However, there is room for improvement.

Major comment:

I see that the authors of this study are frequently using passive voice in writing the paper. I think that the **passive voice** is weak and sometime incorrect. I would suggest using active voice in some sections of the paper.

**Minor comments:**

**INTRODUCTION**

1) “In terms of the latter, there is some evidence suggesting a link between a sedentary lifestyle and the development of chronic pain”

I would suggest talking about lower levels of physical activity or inactivity, which is related to your topic, instead of talking about sedentary behavior. “Sedentary behavior is any waking behavior characterized by an energy expenditure ≤1.5 metabolic equivalents (METs), while in a sitting, reclining or lying posture”. Therefore, it is likely for someone to accumulate large amounts of both vigorous physical activity and sedentary behavior in one day. For example, someone may work 8 hours on desk and in the same day he may run or swim for 2 hours.

I would recommend you reading these articles: <https://ijbnpa.biomedcentral.com/articles/10.1186/s12966-017-0525-8>

<https://www.nrcresearchpress.com/doi/10.1139/h2012-024#.XpWFA8gzaUk>

I would suggest for you to rewrite this paragraph and focus on studies that measured the association between physical activity levels and pain.

**METHODS**

1) Procedure

The procedure is confusing me! As I see that you are talking about a different study published in 2018! I was expecting to find some information on the procedures that you followed in your study. I think you need some extra sentences in the beginning of this section before starting to talk about SHoT2018.

2) Physical exercise

“In the current study, the response options “Never” and “Less than once a week” were combined for the frequency item, constituting the reference category.”.

As I see in the results section that the highest physical activity category was set to be the reference category. Therefore, I see conflicted information in this study! Could you clarify that?

I would suggest that you set the lowest physical activity category to be the reference category. It is very important for the readers and researchers to look at whether increasing physical activity is associated with any additional benefits for chronic pain.

3) Sociodemographic information

“economically active” (annual income > 10,000 NOK) versus “economically inactive” (< 10,000 NOK).”

So, what about people who will have income equal to 10,000! They will be categorized as economically active or inactive?

4) Sleep duration

What do you mean with rise time? Do you mean wake time?

**RESULTS**

1) In general, I suggest for you to set up the lowest category to be the reference category.
2) “Table 1. Sociodemographic and clinical characteristics of the SHoT 2018 study.”

I would suggest for you talking about the data that you used in your study. You could only clarify in the procedure section that you used data from the SHoT2018. Could you do that throughout the manuscript?

3) Physical exercise and chronic pain

I see that in this section that you are sometimes talking about chronic pain intensity and sometimes chronic pain locations! However, when I looked at the table I found “chronic pain” only! Could you be clearer in your writing about this point?

4) Physical exercise and moderate to severe chronic pain

“Also, the ORs for exercise duration and moderate-to-severe chronic pain among males were higher than those found for chronic pain.”

I did not see big differences between them! I would suggest for you to focus on the significant results.

**DISCUSSION**

1) “This large national health survey from 2018, inviting all Norwegian full-time college and university students in the age 18-35, has several interesting findings.”

It does not make sense to me whether you are talking about your study findings or the survey!

2) “The observed prevalence of chronic pain in the current study was high but comparable to what

has also been observed in similar studies. In a recent review and meta-analysis of chronic pain in

epidemiological studies, a pooled chronic pain prevalence of 31% was reported, although the authors concluded that the lack of consistency in defining chronic pain makes evaluations and comparisons across study populations difficult”.

The prevalence of chronic pain in your study was around 54%! I do not think it is similar to the review that your reported which says 31%!

3) “Strengths of the SHoT study include the very large sample size, in combination with several well-validated questionnaires.”.

I think this sentence is related to the next paragraph!

4) Could you write a conclusion paragraph at the end of the DISCUSSION section?
